# Supplementary material for: An efficient and stable solar flow battery enabled by a single-junction GaAs photoelectrode
Source: Nat Commun. 2021 Jan 8;12:156. doi: 10.1038/s41467-020-20287-w (PMC7794367; doi:10.1038/s41467-020-20287-w)
Supplement: Supplementary file 1 — Supplementary Information [file 41467_2020_20287_MOESM1_ESM.pdf]

Supplementary Information

An efficient and stable solar flow battery enabled by a single-junction GaAs photoelectrode

Hui-Chun Fu,[1,2$], Wenjie Li[1$], Ying Yang[1,3$], Chun-Ho Lin[2], Atilla Veyssal[1], Jr-Hau He[2,4✉], Song Jin[1✉]

[1]Department of Chemistry, University of Wisconsin-Madison, 1101 University Avenue, Madison, Wisconsin 53706, USA.

[2]Division of Computer, Electrical and Mathematical Sciences and Engineering, King Abdullah University of Science and Technology, Thuwal 23955-6900, Saudi Arabia.

[3] Shaanxi Provincial Key Laboratory of Electroanalytical Chemistry, Key Laboratory of Synthetic and Natural Functional Molecule Chemistry of the Ministry of Education, College of Chemistry & Materials Science, Northwest University, Xi'an, 710127, China.

[4]Department of Materials Science and Engineering, City University of Hong Kong, Kowloon, Hong Kong, China.

✉email: jrhauhe@cityu.edu.hk; jin@chem.wisc.edu

[$]Hui-Chun Fu, Wenjie Li, and Ying Yang contributed equally to this work

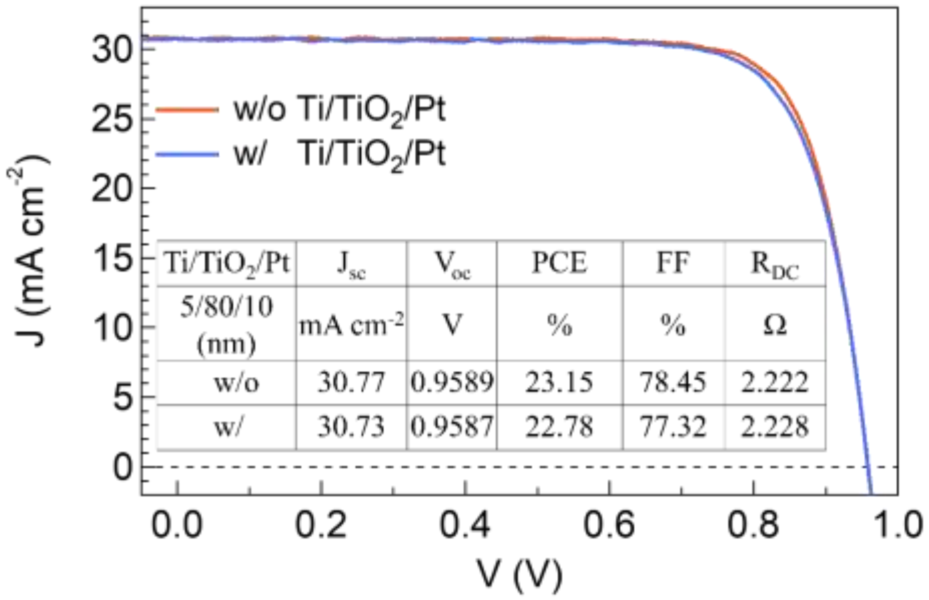

Supplementary Figure 1. J-V performance of the solid-state SJ-GaAs cells. J-V curves are shown for the SJ-GaAs cells with (blue) and without (red) the $Ti/TiO_2/Pt$ (5/80/10 nm) protection layer on the solar cell surface and the performance of these two solar cells is summarized in the inset table.

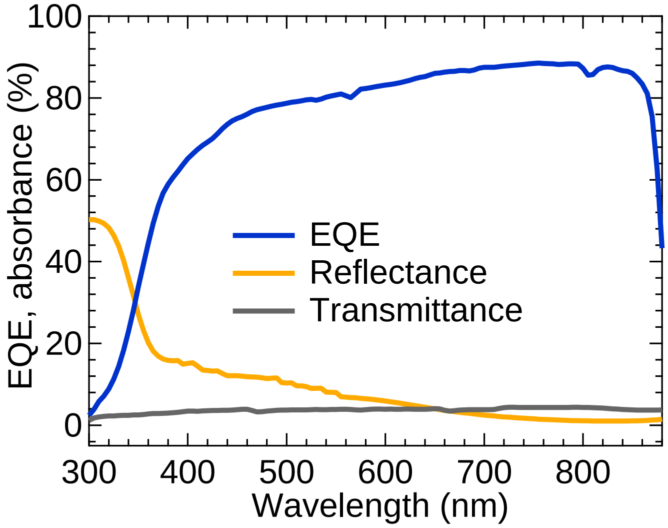

Supplementary Figure 2. Optical and electrical performance of the GaAs cell. Reflectance (organe), transmittance (black) and EQE (blue) spectra of the solid-state SJ-GaAs cell.

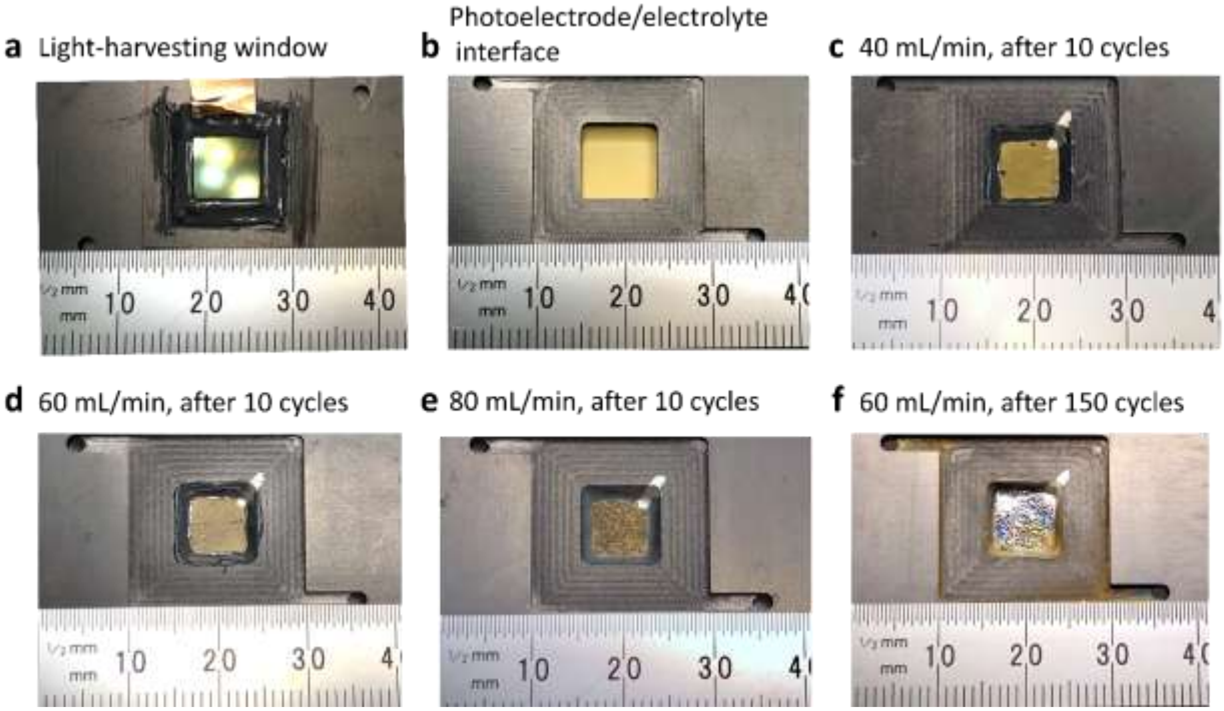

Supplementary Figure 3. Photographs of the SJ-GaAs photoanode. a Light-harvesting window. **b** Photoanode/electrolyte interface. Photoanode/electrolyte interface before and after 10 SFB charging/discharging cycles at the flow rates of **c** 40 mL min^{-1}, **d** 60 mL min^{-1}, **e** 80 mL min^{-1}, and **f** after 150 SFB charging/discharging cycles at a flow rate of 60 mL min^{-1}. The concentration of active redox couples were 0.2 M in all the cycling tests.

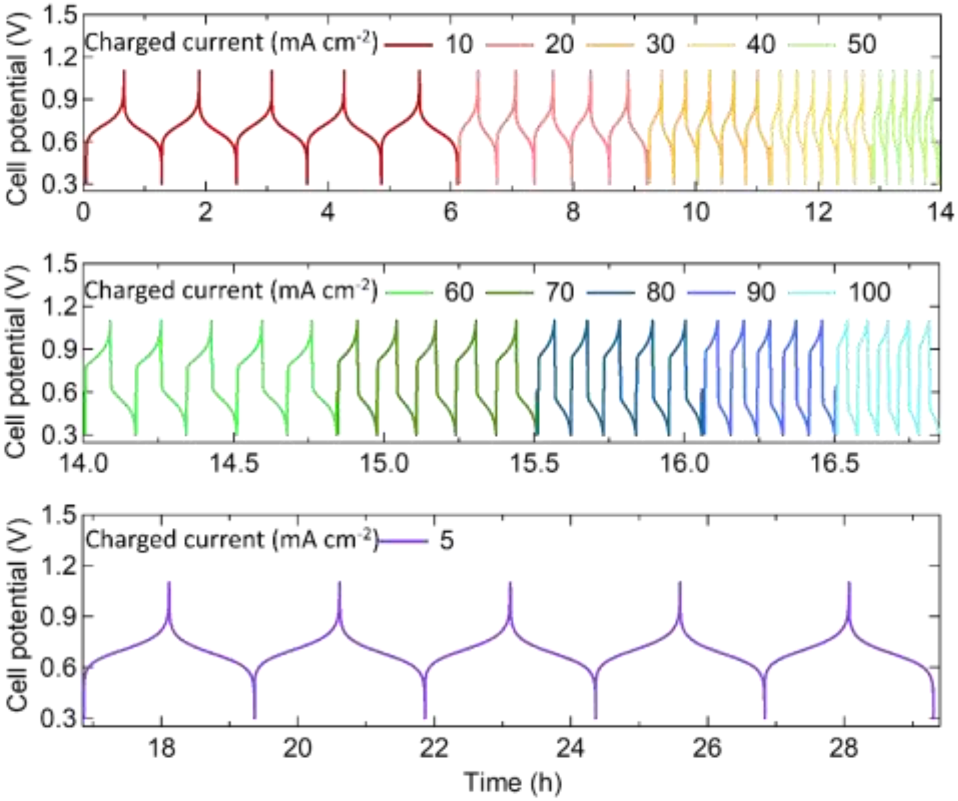

Supplementary Figure 4. RFB cycling performance using BTMAP-Vi/BTMAP-Fc redox couples. Representative cell potential profiles with respect to time of the RFB cycled at different current densities of 5 to 100 mA cm^{-2} with cut-off voltages of 1.1 V and 0.3 V. The redox couple concentrations were 0.20 M.

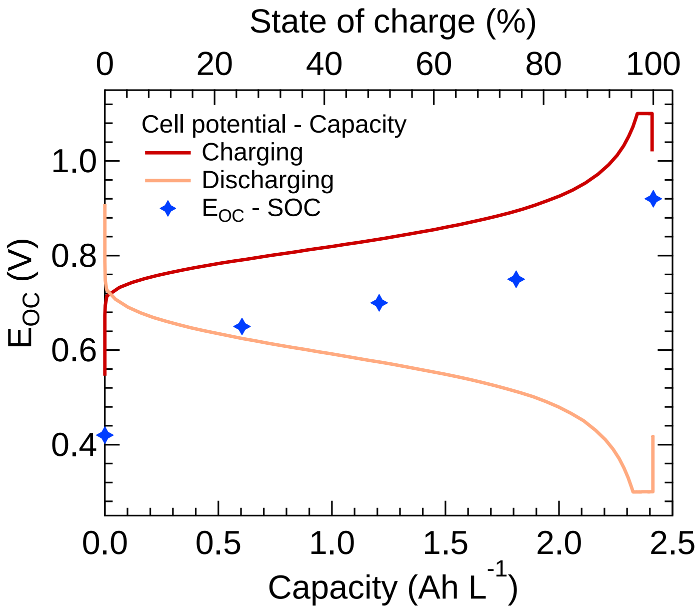

Supplementary Figure 5. Representative cell potential-capacity profiles. A galvanostatic-potentiostatic charge/discharge cycle of the RFB built with 0.2 M BTMAP redox couples. A galvanostatic current density of 50 mA cm^{-2}, cut-off voltages of 1.1 V and 0.3 V and a cut-off current density of 1 mA cm^{-2} were used. The RFB showed a galvanostatic-potentiostatic discharge capacity of 2.41 Ah L^{-1}. The open circuit voltages (E_{OC}) of RFB with respect to SOC are presented by blue stars.

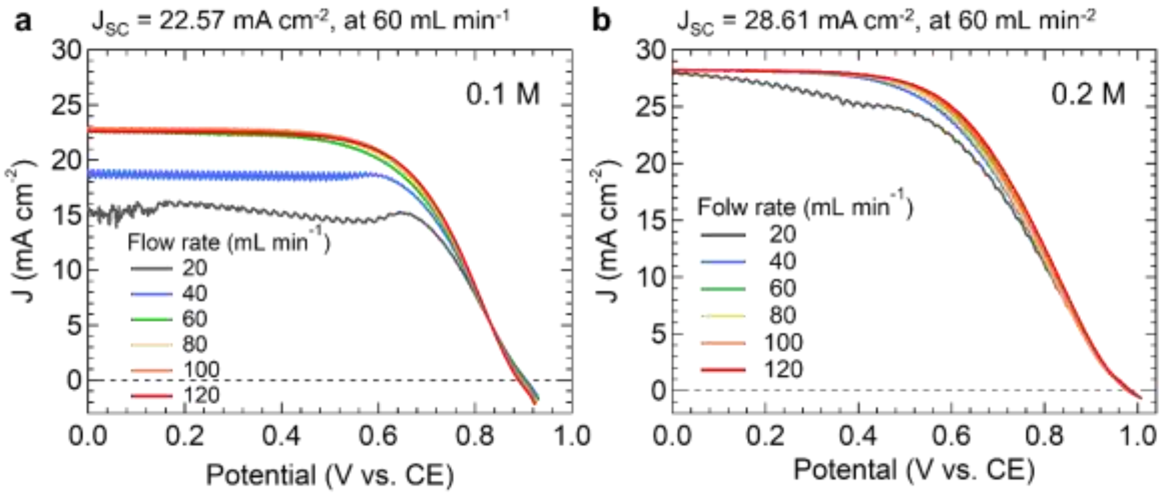

Supplementary Figure 6. LSV performance of the GaAs photoanode. The J-V curves were measured at different electrolyte flow rates and concentrations of BTMAP redox couples. LSV performance of the photoanode in electrolytes with redox couple concentrations of **a** 0.1 M and **b** 0.2 M, measured under one Sun illumination at solar cell mode and flow rates of 20-120 mL min^{-1}.

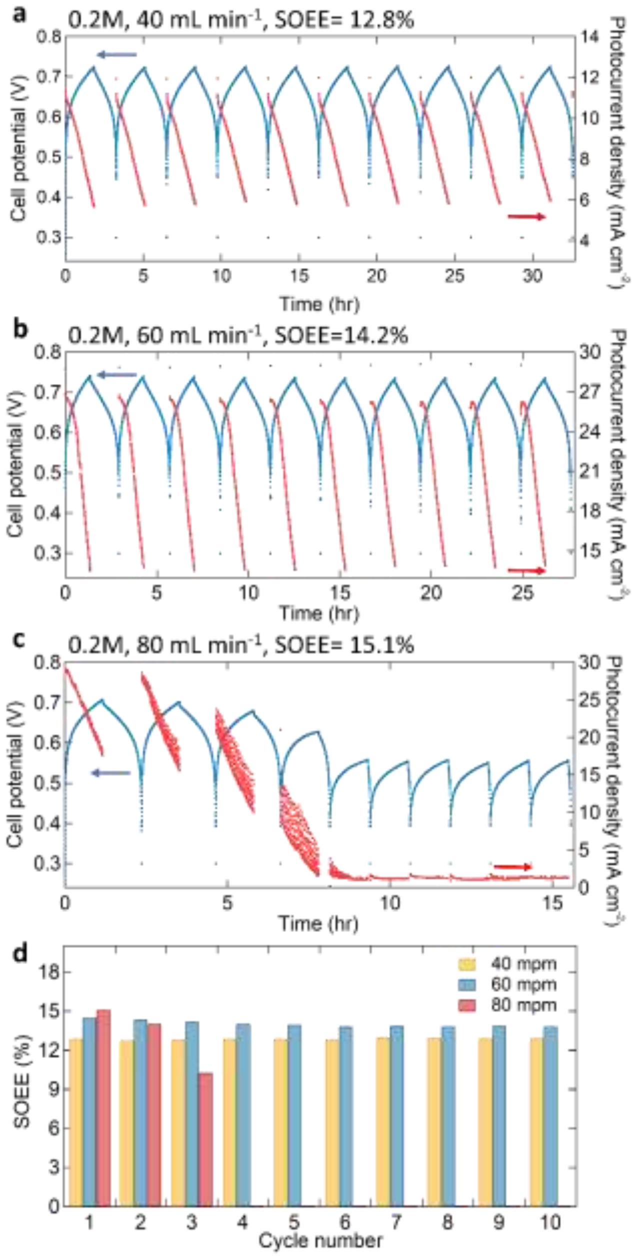

Supplementary Figure 7. Cycling performance of the integrated SFB device. a-c SFB device cycling behaviors at the flow rates of 40, 60 and 80 mL min^{-1} (mpm), showing cell potential between cathode and anode (blue curves), as well as the photocurrent density delivered by the GaAs photoanode (red curves). Each cycle started with a bias-free solar charging process followed by a galvanostatic discharging step at the current of 11 mA until reaching the cutoff potential (0.3 V). **d** SOEE of the SFB device cycled with different electrolyte flow rates of 40 (yellow), 60 (blue), and 80 (red) mpm for 10 cycles. Note that the 0.2 M BTMAP redox couples were used in all measurements.

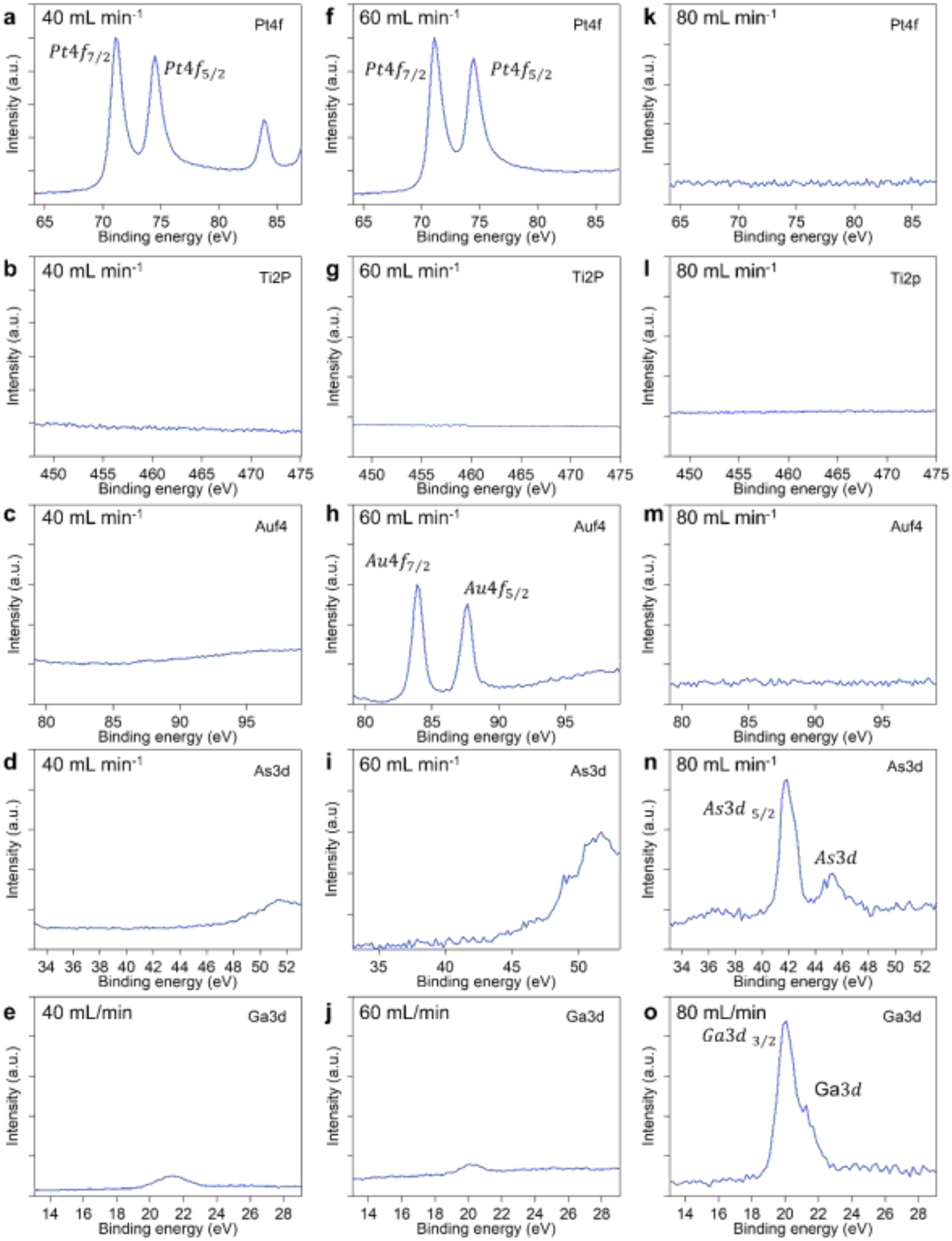

Supplementary Figure 8. X-ray photoelectron spectroscopy (XPS) analysis of the GaAs photoanode. The Pt 4f, Ti 2p, Au 4f, As 3d and Ga 3d peaks of the XPS data collected from the electrolyte contact surface of the GaAs photoanode after 10 SFB charging/discharging cycles at flow rates of **a**-**e** 40, **f**-**j** 60 and **k**-**o** 80 mL min^{-1}.

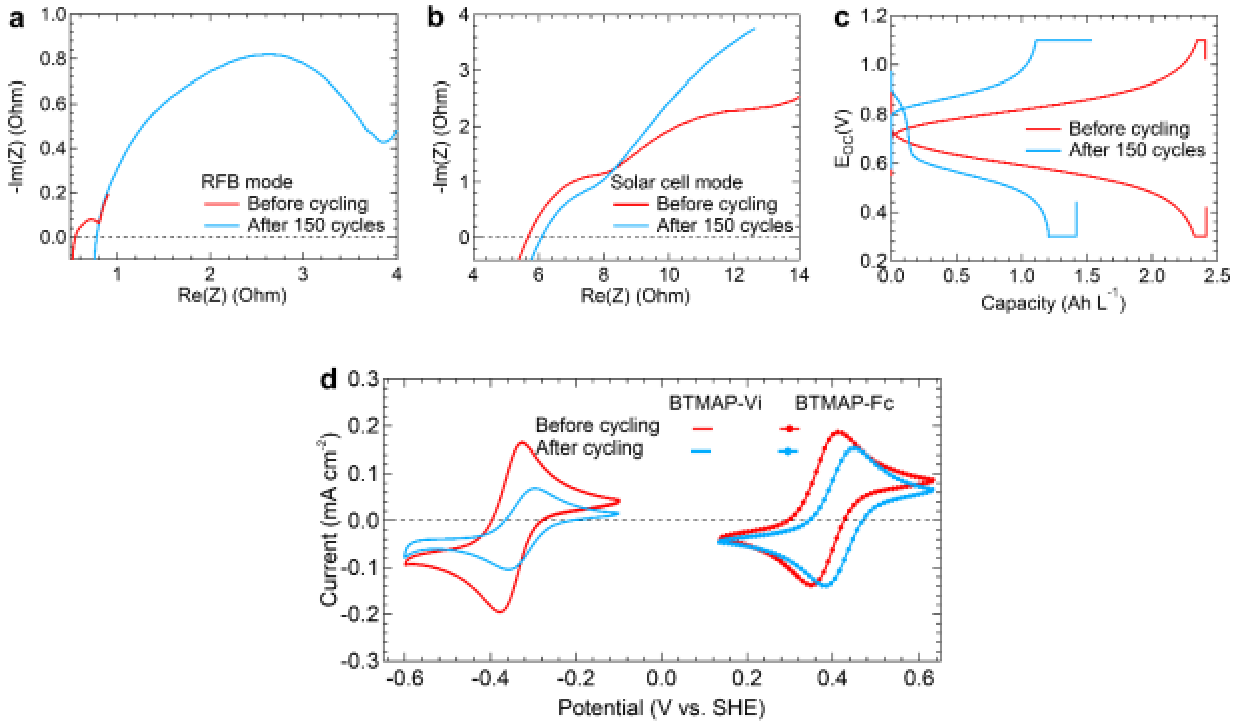

Supplementary Figure 9. Characterization of the SFB device before and after 150 cycles. Electrochemical impedance spectroscopy (EIS) measured under **a** RFB mode and **b** solar cell mode, which revealed the increased cell resistance after the cycling. **c** Galvanostatic-potentiostatic for capacity determination of the SFB device. A capacity of 1.41 Ah L^{-1} can be retained after 150 cycles. **d** Before and after 150 SFB cycles of the cyclic voltammogram of BTMAP-Vi and BTMAP-Fc. Note that, the concentration of each redox couple was 5.0 mM in 1.0 M NaCl for separate experiments and the scanned rate at 10 mV s^{-1} on a glassy carbon electrode during the measurements.

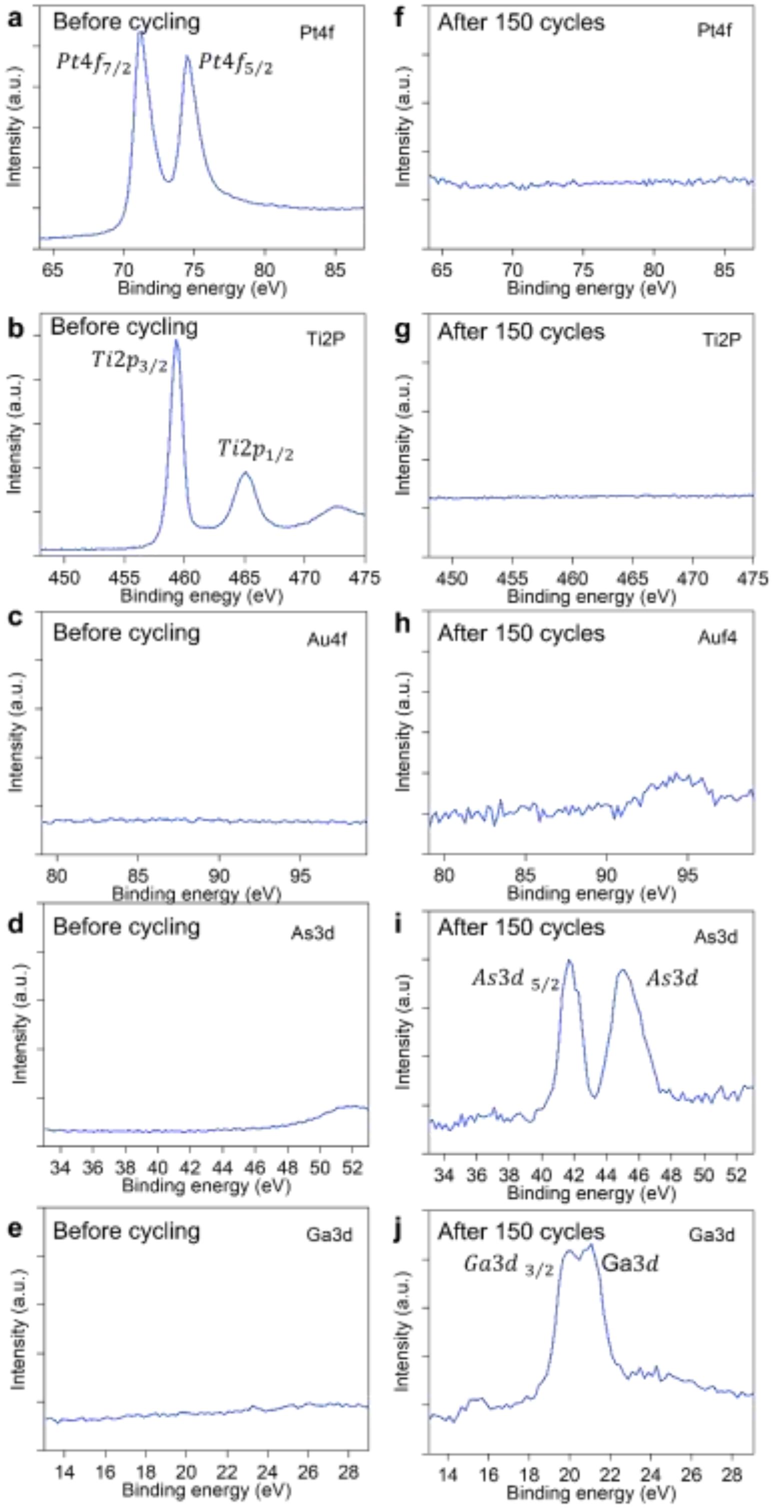

Supplementary Figure 10. XPS of the GaAs photoanode before and after 150 cycles. The Pt 4f, Ti 2p, Au 4f, As 3d and Ga 3d peaks of the XPS data were collected on the electrolyte contact surface of the GaAs photoanode before **a**-**e** and after **f**-**j** 150 SFB cycles.

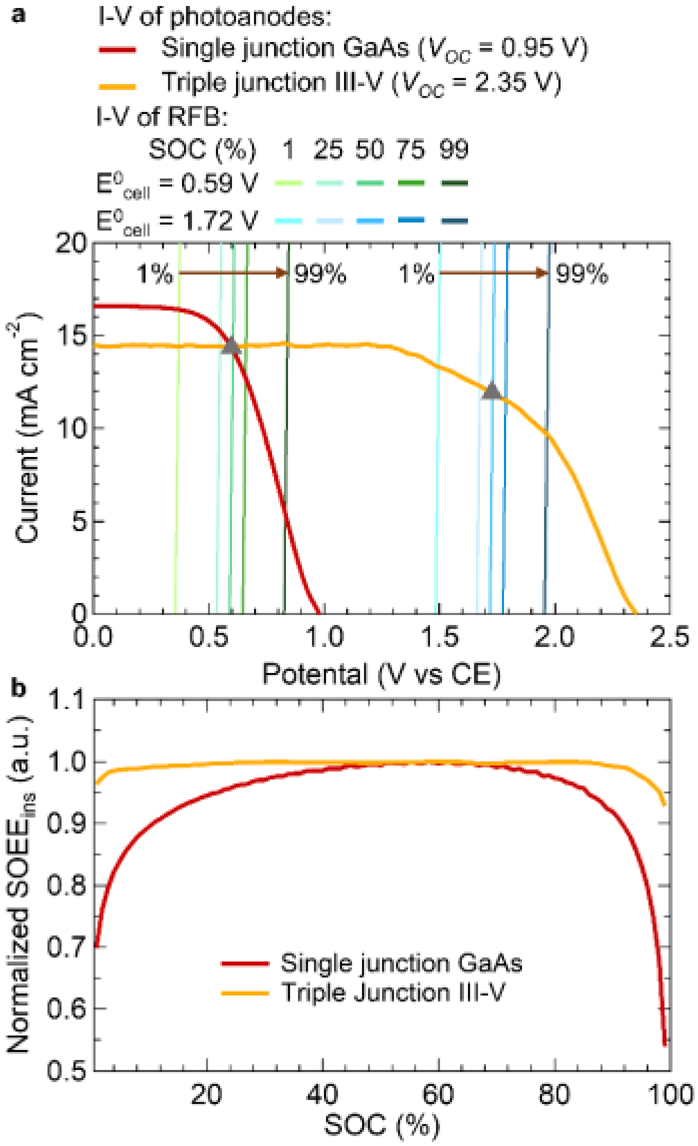

Supplementary Figure 11. Voltage mismatch and SOC swing profiles of the photoelectrodes. Overlaid hypothetical current-potential behaviors between RFBs and two different photoelectrodes with different V_{oc}. **a** J-V performance of the SJ-GaAs (red) and triple junction III-V (orange, reproduced from Ref.15. DOI: 10.1016/j.chempr.2018.08.023) photoanodes measured at solar cell mode overlaid with the I-V cures of RFBs at different SOC simulated with the respective optimally matched E^0_{cell} of 0.59 V (green lines) and 1.72 V (blue lines). The SJ-GaAs photoelectrode was measured at the flow rate of 60 mL min^{-1} under one Sun illumination at 50% SOC. **b** Normalized $SOEE_{ins}$ as a function of SOC for both photoelectrodes.

In Supplementary Fig. 11a, the V_{MPP} of the SJ-GaAs and triple junction III-V photoelectrodes of 0.61V and 1.73 V (marked triangles) can be obtained from the I-V curve of each cell, respectively. By overlaying the I-V curves of the RFB at the different SOC (1 to 99% SOC), the swing range of E_{cell}(SOC) by the matched E^0cell of 0.59V (for SJ-GaAs) and 1.72V (for triple junction GaAs) can be mapped. The SOEE of the SFB is very sensitive to the LSV behavior of the photoelectrode, the decreased FF of the GaAs photoanode would significantly alter its voltage matching with the redox couples in SFBs (see the red curve in Supplementary Fig. 11a). In contrast, the same SOC swing will not create as much in mismatch in higher V_{OC} photoelectrodes such as tandem III-V cell (see the orange curve in Supplementary Fig. 11a), because of the smaller relative voltage shift through the J-V curve. Accordingly, the instantaneous SOEE is much less sensitive to the SOC for a higher Voc photoelectrodes (Supplementary Fig. 11b), therefore tandem photoelectrodes with higher Voc are more likely to achieve better voltage match and higher SPUR of the SFB.

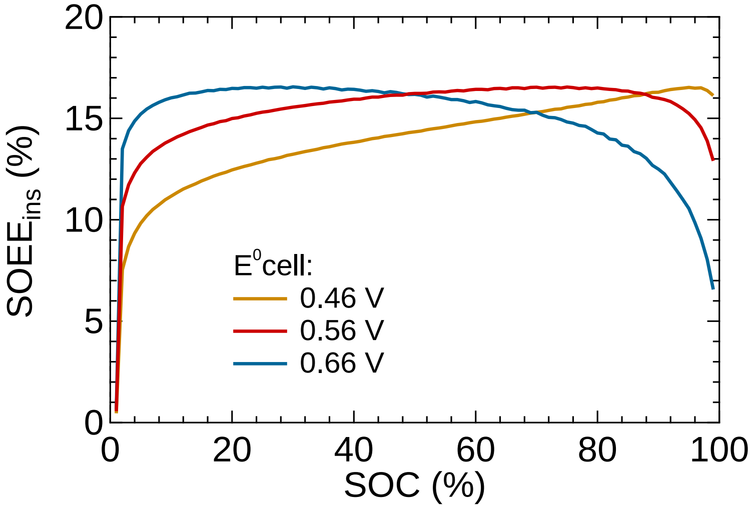

Supplementary Figure 12. The simulated SOEE$_{ins}$-SOC curves. The simulation results were calculated by the I-V characteristic of SJ-GaAs photoanode at the E^0_{cell} of 0.46, 0.56 and 0.66 V.

According to the SOEE$_{ins}$-SOC curves, the charging behavior of the SJ-GaAs SFB by using the E^{0}cell of 0.46 V (less than optimized E^0_{cell}, yellow curve), 0.56 V (the optimized E^0_{cell}, red curve) and 0.66 V (higher than optimized E^0_{cell}, blue curve) can be predicted. The steady SOEE$_{inc}$ of SJ-GaAs SFB over the 1 to 99% SOC when the SFB was integrated the redox couple with the optimized E^0_{cell}.

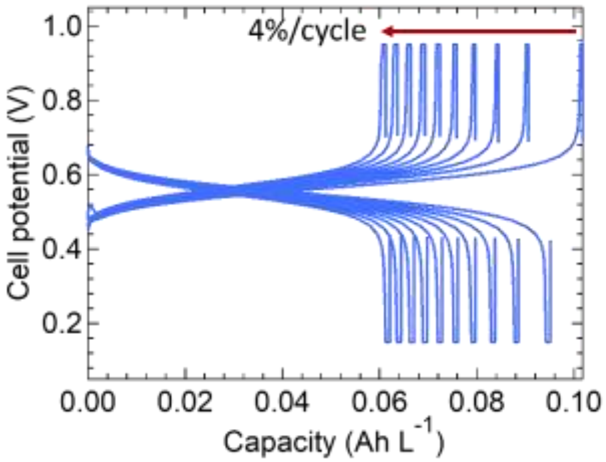

Supplementary Figure 13. The galvanostatic-potentiostatic charge/discharge cycling of the RFB. A significant capacity fade of 4% per cycle could be observed. The concentration 0.05 M was used for both BTMAP-Fc and N^{Me}-TEMPO in 1.0 M NaCl supporting electrolyte.

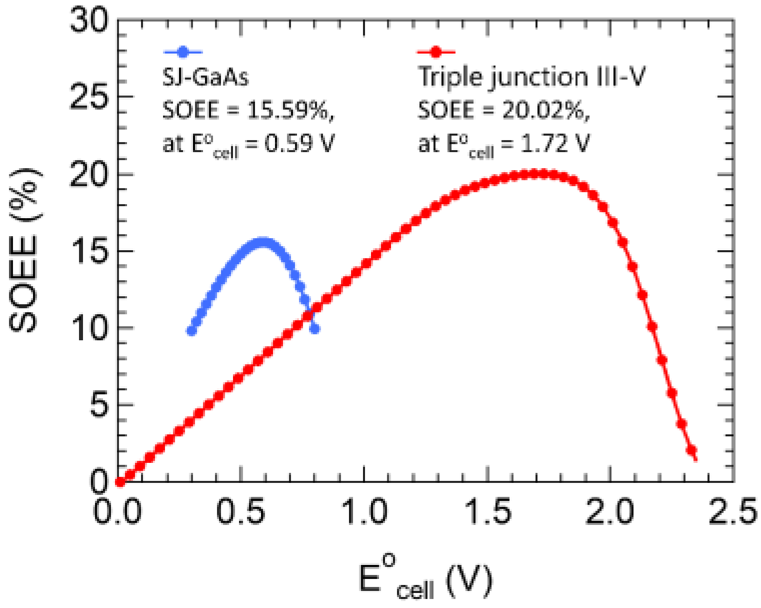

Supplementary Figure 14. Simulated SOEE as a function of SFB cell potential (E^0_{cell}). The optimal E^0_{cell} of 0.59 and 1.72 V were determined according to the simulations for single-junction GaAs (blue) and triple-junction III-V solar cell (red), respectively. The E^0_{cell} of 1.25 V was used in the previously reported SFB based on triple-junction III-V solar cell (ref. 12).
